# The interaction between electromagnetic fields at megahertz, gigahertz and terahertz frequencies with cells, tissues and organisms: risks and potential

**DOI:** 10.1098/rsif.2017.0585

**Published:** 2017-12-06

**Authors:** Sergii Romanenko, Ryan Begley, Alan R. Harvey, Livia Hool, Vincent P. Wallace

**Affiliations:** 1 School of Physics, The University of Western Australia, Perth, Western Australia, Australia; 2 School of Human Sciences, The University of Western Australia, Perth, Western Australia, Australia; 3 Perron Institute for Neurological and Translational Science, Perth, Western Australia, Australia; 4 Victor Chang Cardiac Research Institute, Darlinghurst, New South Wales, Australia

**Keywords:** electric field, millimetre wave, terahertz radiation, DNA, cell, tissue

## Abstract

Since regular radio broadcasts started in the 1920s, the exposure to human-made electromagnetic fields has steadily increased. These days we are not only exposed to radio waves but also other frequencies from a variety of sources, mainly from communication and security devices. Considering that nearly all biological systems interact with electromagnetic fields, understanding the affects is essential for safety and technological progress. This paper systematically reviews the role and effects of static and pulsed radio frequencies (10^0^–10^9^ Hz), millimetre waves (MMWs) or gigahertz (10^9^–10^11^ Hz), and terahertz (10^11^–10^13^ Hz) on various biomolecules, cells and tissues. Electromagnetic fields have been shown to affect the activity in cell membranes (sodium versus potassium ion conductivities) and non-selective channels, transmembrane potentials and even the cell cycle. Particular attention is given to millimetre and terahertz radiation due to their increasing utilization and, hence, increasing human exposure. MMWs are known to alter active transport across cell membranes, and it has been reported that terahertz radiation may interfere with DNA and cause genomic instabilities. These and other phenomena are discussed along with the discrepancies and controversies from published studies.

## 1. Introduction

Organisms, though electrically neutral, contain charged ions, polarized molecules and electric fields which obey the laws of electromagnetism and thermodynamics. For example, every cell possesses a resting transmembrane potential, and the absence of such a potential is clear evidence of a non-functional (dead) cell. In neurons, electrical impulses travel along the cell to transfer signals; electrical excitation of muscle cells leads to mechanical contraction (and, in the case of, cardiomyocytes, synchronization of excitation), and transepithelial potential determines the resistance and integrity of tissue [1]. These are just some examples of the role of electromagnetism in physiology.

The existence of an electrical potential across a cell membrane sustains a chemical gradient between intra- and extracellular spaces and this drives transmembrane transport of organic (e.g. glucose) or inorganic molecules and ions. It is also a driving force for different types of transmembrane currents which could result from carrier-specific or non-specific conductance and thus have vastly different biological effects. For example, the inward sodium and calcium transmembrane currents have the same directionality and could be of the same magnitude, but calcium currents will also cause the depletion of endoplasmic reticulum (and ‘self-amplification’ of the calcium signal) and trigger a number of secondary biochemical reactions. The kinetics of ion channel permeability defines the shape of an action potential (AP) curve as well as the firing rate. For example, the rapidly inactivating A-type potassium channel is one of the key determinants of the AP spiking rate [2], whereas the leak current due to two-pore-domain potassium controls the resting membrane potential [3]. This, together with the kinetics of the sodium, chloride and calcium channels, results in diverse and complex electrochemical mechanisms of cellular regulation that influence short- and long-term physiological phenomena. All these energy-dissipating processes are possible due to the ongoing synthesis of ATP by mitochondria (partly due to the existence of the mitochondrial potential). The physiology of the membrane is also a determinant of long-term effects, for example permanently open TRP channels may cause excessive calcium elevation in a cell and cause excitotoxicity and cell death.

Despite the importance of electric fields and associated flux of various charged atoms and molecules as well as the translocation of polar molecules in the life cycle of any cell and living organism, only a fraction of the electromagnetic spectrum is employed by nature. Indeed, the static (resting membrane potential) and alternating (e.g. AP) electric field is in the range of only a couple kilohertz. However, there are theoretical predictions of the existence of megahertz to terahertz oscillations in the living cells made by Fröhlich [4]. The existence of longitudinal electric modes in biological systems was based on unique dielectric properties of cell membranes (which are capable of sustaining electric field gradients of approx. 70 mV across a 4 nm membrane bilayer) and weak molecular bonds, such as hydrogen bonds, and long-range interactions. However, attempts to show the existence of such electric modes in cells are somewhat controversial (vide infra).

**Figure 1.**
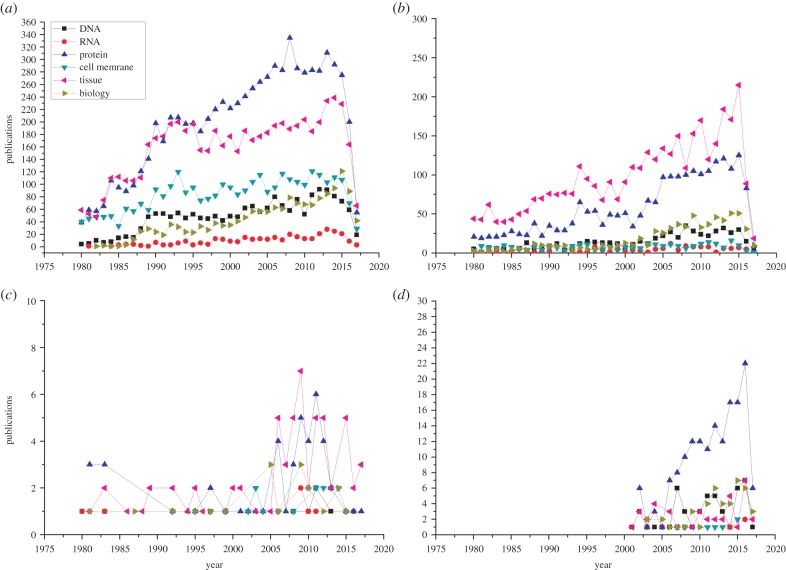
The progress in research measured as number of publications studying the effect of an electric field (*a*), radio waves (*b*), MMWs (*c*) and terahertz radiation (*d*) on various biology samples (DNA, RNA, proteins, cell membranes, tissues and other biology). The data are taken from the PubMed portal (www.ncbi.nih.gov). (Online version in colour.)

Other physiological mechanisms include the multiple intra- and intermolecular interactions, both short and long range, and energy transfers within assemblies of biomolecules and between biomolecules and their environment. A complex amino acid sequence has a tremendous amount of possible configurations because every amino acid can assume different orientations with respect to its neighbours. However, because almost every protein contains hydrophobic and hydrophilic domains and due to the polar nature of the comparatively small water molecules, the tertiary protein structure tends to form a hydrophobic core inaccessible to water. In this way, about 80% of all hydrophobic side chains are hidden, resulting in the most favourable entropy [5,6]. About half of the protein-associated water interacts with the protein backbone. Some of these water molecules are trapped in internal cavities of the protein globule, and the rest are associated with side chains. This water density is about 10–20% higher than free molecules [7] and creates predispositions for more intense long-range interactions, collective vibrations and transitions. These water vibrations are the basis for interaction with rapidly alternating electromagnetic fields, whose characteristic frequencies span a wide range from several megahertz (including millimetre waves (MMWs)) and up to the terahertz part of the electromagnetic spectrum. Studies dedicated to finding the role and importance of collective interactions in biological systems became the subject of scientific interest comparatively recently (figure 1). It would be unfair though to say that no attempts have been made before. Some studies have considered complex inter- and intramolecular interactions on the basis of many nonlinear biological phenomena [8], some of them even had a reasonable theoretical basis [9,10], but such studies are infrequent, often narrow in scope, and do not provide a comprehensive picture of physiology. Recent advances in computational biology, molecular dynamics and improved instruments have given a new momentum to studies at a subcellular but supra-molecular scale. Also, a good understanding of these phenomena also promises improved biomedical applications in diagnostic and therapeutic technologies.

## 2. The homeostatic role of electromagnetic fields

As mentioned above, some types of electric/magnetic fields support the cell and organism functionality and adaptability. Those internal field forces are equal players along with the most crucial molecules in the cell, like DNA, proteins and lipids. Examples of intrinsic electromagnetic forces are reviewed in this section.

The electromagnetic characteristics (or status) of the cell are one of the driving forces in the cell life cycle. For example, it is well known that mature neurons do not proliferate. Thus, deceased neurons are generally not replaced (it is worth mentioning that contemporary studies in neurogenesis have found exceptions to this rule in some specific regions of the adult central nervous system, including in humans) and also have one of the highest transmembrane potentials (−50 to −80 mV). By contrast, cancer cells have a tremendous potential to proliferate and also commonly have a very low (−30 to 0 mV) transmembrane potential. Interestingly, some types of chloride channels are important players in the genesis of cancer [11]. Such a correlation is not coincidental; hyperpolarization of vascular endothelial cells indicates arrest in their cell cycle [12] and the same effect is observed following blockage of potassium transmembrane conductivity in lymphocytes, Schwann cells and astrocytes. Vice versa, a wide variety of potassium channels and especially voltage-sensitive channels are active during G1/S transition (gene replication stage of the cell cycle) [13], and their activity could be accompanied by the activity of some types of calcium and chloride channels.

Another example of the vital role of transmembrane potential is the regulation of nuclear factors (erythroid-derived 2) such as 2 (Nrf2); activation of the Nrf2 antioxidant response signalling pathway controls the expression of a protein involved in defence against oxidative or electrophilic stress [14]. It has been shown that depolarization of the cell membrane can affect the local activity of the Nrf2 transcription factor [15]. One study revealed a striking ability for mature neurons *in vitro* to increase RNA synthesis following an induced reduction in the transmembrane potential of as short as 1 h duration. With a prolonged transmembrane potential depolarization of 3 h, corresponding changes to DNA synthesis were also observed [16]. A more recent study [17] revealed that transmembrane potential is one of the key regulators of aggressive cancer tumour growth mediated by nanoscale reorganization of phosphatidylserine and phosphatidylinositol 4,5-bisphosphate but not other anionic phospholipids of the cell membrane, which eventually regulate K-Ras signalling and drive cell division.

Electromagnetic effects are not only observed at the single cell level but also on tissues, and even have a role in ‘non-excitable’ tissues. For example, across the frog epidermis, there is a relatively large electric gradient of about 100 mV. Frogs have a so-called ‘transporting epithelium’ which consists of multiple cell layers horizontally connected by tight junctions. The skin's outer layer absorbs sodium ions from the external environment which diffuse to the deeper basolateral membrane and are then pumped into the interstitial fluid by Na/K-ATPase; the result is a net flux of sodium ions into the body and formation of a transepithelial potential. Following skin damage, this transepithelial potential drives longitudinal currents in the extracellular space and creates the gradients needed for directional cell migration, proliferation and healing. The transepithelial potential is also essential for guinea pig skin and human skin; where it reaches up to 140 mV mm^−1^. In the case damage to the epidermis, a steady electric gradient persists at the lesion edge. It is essential for wound healing in embryogenesis [18] and tissue recovery after mechanical damage. For example, prolonged depolarization of the spinal injury site in salamander prevented axon regeneration [19]. Importantly, embryonic development in the presence of an externally applied electric field can cause developmental abnormalities; and is dependent on the intensity and direction of the field [18]. The effect was observed in chick embryos when the internal electric field pattern was deteriorated with tiny capillary shunts; a one-third decrease in current amplitude flowing through the posterior intestinal portal resulted in more than 90% embryo abnormalities [20]. Moreover, there is evidence that electric field patterns may be involved in the regulation of normal development of left–right body asymmetry [21]. The spatial regulation of gene expression occurs in relatively large cellular assemblies and thus requires reliable and fast synchronization that sometimes cannot be successfully done with via intercellular transport of endogenous molecules [22]. Transcellular fields and current flow via tight junctions (any of its variations) are used to facilitate this task. In a chick embryo in the stage of 2–4 left–right asymmetry, a voltage gradient in the epithelial membrane potential was found to be as large as 20 mV. It was shown that this potential gradient depends on H/K-ATPase activity and a pharmacological block of this pump perturbed gene expression on both sides of the embryo. For more detail on this topic, the reader may refer to the excellent review by McCaig [21]. The important conclusion at this stage is that endogenous electromagnetic fields are diverse in their intensities, spatial and temporal patterns, biological effects and tissue distribution. They are essential to many processes in developmental stages as well as in mature cells, tissue, organs and the whole organism [23]. There is a growing body of evidence that biological organisms are especially sensitive to electromagnetic fields during periods of development.

## 3. The interaction of external electromagnetic fields and tissues

Scientific interest in the effect of externally applied electromagnetic fields on different tissues and cells began in the mid-twentieth century [24,25]. When cultivated dorsal root ganglia neurons were exposed to an external static electric field, it stimulated the formation of new protrusions and neurites. In experiments with neurons and neuroblasts of both vertebrate and invertebrate origin, an external electric field caused the preferential growth of neurites towards the negative electrode, the rate of growth was found to be significantly higher towards the cathode, and in some cases, existing neurites changed their direction of protrusion [26,27]. Robinson [20] provides some examples of exogenous static electric field effects on different cell types in different species, which indicates that sensitivity to an exogenous electric field is a universal and omnipresent phenomena in biology. For example, it was shown that neural crest cells of Xenopus and Ambystoma (axolotl) and quail migrate towards a cathode; and the same happens with fibroblasts, epithelial cells and fish scales, and that Xenopus embryo and myoblasts tend to align perpendicular to the direction of the field [20,26]. The notable aspect of all these reports is that the cell reaction in all cases was observed for electric field intensities below 1 mV cell^−1^ diameter, which is not a significantly large when compared with the resting membrane potential.

It is clear that biological objects interact not only with static fields but also with alternating electromagnetic fields at a range of frequencies. The biological response is varied and depends on the organism, tissue, molecular composition of the particular cell as well as on the parameters of the electromagnetic field itself: frequency, intensity, modulation, polarization, pulsing mode, instantaneous and average power, total energy absorbed etc. There is evidence of dynamic changes in the electromagnetic properties of tissue due to its natural activity, which alters the reaction of biological objects to externally applied electromagnetic radiation. It was shown in crab nerves *in vitro* that due to alterations in the transmembrane potential in the neuronal membrane, correlative changes in (externally applied) light scattering were observed along with the birefringence effect [28]. The same effect was observed in later studies with a squid giant axon and a correlation between optical retardation and externally imposed voltage steps on the axon membrane was reported; the authors assumed that effect might originate from molecular relaxation processes analogous with the Kerr effect [29]. Overall, the above evidence shows that electromagnetic effects are ubiquitous in physiology, yet the precise nature of these effects varies significantly.

### 3.1. Types of interaction

Application of an external electromagnetic field to biological objects induces an absolute and relative redistribution of internal charges with respect to the field lines. This time-consuming process is characterized by ‘relaxation’ time of the system. There are different types of relaxation, which define material properties and are expressed upon application of alternating electric fields. The most common are dipole relaxation and ionic, atomic and electron polarization, and they are ordered according to their resonant frequencies. The dominance of any one of these types of relaxation in a whole system depends on the frequency of the external stimulus. In reality, many other types of relaxation are observed, such as boundary effects and complex molecular vibrations. In the simplest model, external fields cause alignment of the molecules along their tension lines, and as the field direction alternates molecules follow these changes. At some point, the frequency of the alternating field is so rapid that molecules or any other field-sensitive entities cannot follow the directionality change. This is a key moment which happens when external field frequency matches the relaxation time of the system. Such system behaviour is called the simple Debye model, and it is a simplistic representation of the actual process. Representation of the dielectric permittivity is a complex form of the real and imaginary part which allows one to describe the fundamental properties of tissue to accumulate and/or dissipate the energy of electromagnetic irradiation. Owing to the fact that biological tissues are a very complex material consisting of thousands of molecules with different dielectric properties and have rather periodic but not regular structure, it is expected that the dielectric spectrum of tissues will be very complex, with any resonant peaks of any particular component smeared by the irregularity of the structure and contaminated by other components of the sample [30]. Nevertheless, some types of relaxation are very typical for biological samples. For example, polar molecules have more than one relaxation time and this is described by the improved ‘Cole–Cole relaxation’ model. After some experimental work, and to characterize the non-uniform distribution of relaxation mechanisms, the improved ‘Cole–Davidson relaxation’ model was suggested. The next evolutionary step was characterization of the complex polymers and polymer-like molecules which yielded the development of the ‘Havriliak–Negami relaxation’ model. For adequate characterization of the change in the real part of the dielectric permittivity caused by local intermolecular interaction (e.g. solute and solvent), the Kirkwood correlation parameter was introduced into the model [31]. The dielectric properties of the interface between cell membranes and the intra/extracellular solutions can be well described by the Maxwell–Wagner model [31].

In general, from dielectroscopy, biological samples can be characterized as anisotropic, inhomogeneous, polydomain and high dispersion [32]. Altogether this presumes a very irregular and inhomogeneous dielectric spectrum of different biological tissues. In reality, it appears not to be the case. Multiple studies conducted in the range of 10 Hz to 20 GHz (in some cases up to 100 GHz) on different preparations of animal and human tissue samples revealed similar frequency dependence of dielectric parameters. In a fascinating study by Gabriel *et al*. [33], the frequency dependence of permittivity and conductivity (or the real and imaginary part of full dielectric permittivity) for blood, bone, fat tissue, brain grey and white matter, kidney, spleen, heart, liver and skin have been provided [34]. In all cases, the real part of the permittivity started at quite high values (as large as 10^7^) with a gradual but non-monotonous decrease, resembling a superposition of multiple sigmoidal functions, where each sigmoidal function corresponds to a particular type of relaxation process [35]. The step-like changes in dielectric permittivity along the spectrum are divided into four main relaxation regions, namely *α*, *β*, *γ* and *δ*. The *α* relaxation is associated with a slow relaxation process aka ion relaxation. The *β* relaxation accounts for a relaxation of large biomolecules and Maxwell–Wagner polarization and typically spans the region of 100 kHz. The *γ* dispersion is responsible for relaxation of small, polar molecules of high mobility, most typically water, and covers frequencies above 1 GHz [31].

In the analysis conducted by Gabriel *et al.* [33–35], it is emphasized that data for the same type of tissue have some discrepancies which were caused by differences in experimental conditions. In particular, the bone tissue data differ due to specimen orientation; longitudinal or transverse. The same variation due to sample orientation with respect to the electric field vector was also discovered for muscle tissue, and was both effects were found to be highly anisotropic. There are also significant deviations in data for adipose tissue, which is presumably caused by the high variance in its composition, which dependent on its origin [36], which indicates the importance of the water to lipid/protein ratio for the defining relaxation time.

## 4. Biological effects of millimetre wave

### 4.1. Early findings and safety issues

Since the rise of the radio era in the twentieth century, humanity has experienced a rapid increase in exposure to new radiation sources working in MMW and terahertz spectral range. The evolutionary process has had little opportunity to equip us with immunity to this novel kind of external stimulus.

The earliest concerns arose due to the first radar air defence systems introduced during World War II. These phenomena were investigated in several waves of scientific study, but the proposed mechanisms of action were subjected to strong criticism. In his studies, Frey [37] revealed that utilization of electromagnetic pulses with carrier frequencies ranging from 500 MHz to 9 GHz resulted in acoustic-like perception in human subjects. Of particular interest is that ‘radio-hearing’ was observed even in clinically deaf subjects. Another interesting phenomenon was that experimental subjects claimed a good match with complimenting audio-acoustic stimulus. Moreover, the radio-hearing perception was diminished for spectral components below 5 kHz and augmented for the spectral range beyond the classical 20 kHz. It was claimed that the average power needed to cause auditory effects was as low as 0.4 mW cm^−2^, but it was also specifically stressed that impulse power should be high with a threshold of 275 mW cm^−2^. Later studies also indicated that, with respect to evoking a response in a biological system, the power of the stimulus was more important than the energy dissipated in the system [37–40].

Studies into the biological effects of megahertz, gigahertz and up to terahertz radiation have progressed as the environmental sources of radiation have changed and the respective techniques allowing the measurement of its effect on biology have developed. Concerns surrounding the safety of exposure to radiation at megahertz to terahertz frequencies, particularly those in industrial and domestic applications, have remained a significant motivator in research, particularly at frequencies at hertz to kilohertz and partially megahertz range [41–43]. In the early 1970s, as new compact and powerful RF sources became available, a special report on the biological effects of radiation in the MMW range of 1–100 GHz was conducted at the USSR Academy of Science [44,45]. The report included the work of Devyatkov [46], who conducted a series of studies showing that low-intensity MMW radiation in the range 39–46 GHz applied to a yeast culture promoted the growth of the colony. It was concluded that the MMW effect is frequency-dependent and that the time of exposure is also quite an important modulatory parameter, whereas the applied power had only a weak effect. Another study showed that MMW induced suppression of the haemolytic activity of *Staphylococcus aureus*. A similar suppressive effect was observed in *Escherichia coli* [47]. Experimental cultures were exposed to MMW of low intensity for 1 h (daily treatment), and the procedure was repeated for several days, following which physiological parameters (like the viability test) and sensitivity to antibiotics were assessed. Suppression of biological activity (trophic activity and enzymatic activity) was also observed for other bacteria including *Clostridium sporogenesis* and *Clostridium histoliticum* [48]*.* These cells experienced a decrease in size, alteration in metabolism (in particular, metabolism of acidic amino acids was decreased, whereas no changes for alkali amino acids were observed), and suppression of sporogenesis. Another study indicated that MMW exposure of fruit flies for about 1 h did not affect the activity of the subjects but led to losses in fertility and that the effect was carried over for several generations [49]. In experiments on rats, daily exposure to MMW resulted in depressive behaviour, decreased appetite and permanent fatigue [50–52]. Despite the significant experimental sample size, researchers from other laboratories disputed some of the results and conclusions made by Soviet researchers. One of the most disputed statements is a resonant-like effect at certain frequencies.

### 4.2. Theoretical predictions and related experimental outcomes

Nevertheless, those findings are consistent to some extent with the original Fröhlich theoretical conclusions about the role and importance of MMW and terahertz electromagnetic oscillations in biology. Briefly, the theory suggests the existence of the system of dipole oscillators which are capable of forming the long-range Coulomb interactions [4]. In such an environment, the energy exchange between its elements and also with the heat-bath would occur in quanta of energy (within a limited range of frequencies) and then the entire system ‘gives rise to a branch of Z longitudinal electric modes' [4] with a limited frequency range. Moreover, if such a system has a stable and sufficient supply of energy, then it is possible to reach some steady state which could be far from thermodynamic equilibrium. Such steady state is possible if the system of dipolar oscillators would fall into a single longitudinal mode with long-range phase correlation (aka Bose type condensation). Despite being an energy-consuming system, such metastability provides a great potential for system elastic deformations and could play a pivotal role in cell division. The theory looks quite attractive, especially if we consider that any live cell contains and exist in water solutions and a water molecule is a dipole which exhibits long-range hydrogen bond interaction, the dipole feature is also typical for some lipids which form highly organized membranes, some types of sugars (glycosaminoglycans) and even DNA is polyelectrolyte. Moreover, the processes of cell volume homeostasis, asymmetry of the cell membrane, cytoskeleton structure and DNA repair are energy-consuming and require a continuous energy supply, which draws good parallels with Fröhlich's theory.

One of the recent attempts to verify the Fröhlich theory was the work of Williams *et al.* [53]. In the study, three types of confluent and subconfluent cells cultures [human corneal epithelial cells (HCE-T), retinal pigment epithelial cells (ARPE -19) and human embryonic stem cells (hES07)] were monitored up to 72 h after been subjected to pulsed intense terahertz radiation (broadband coherent emission up to 500 GHz with peak power density up to 2.25 kW cm^−12^). Comparison of the irradiated samples with the control group did not reveal any significant alteration in cell morphology, differentiation, proliferation and attachment. By contrast, the studies by Bock *et al.* [54] conducted on mouse stem cells subjected to long-term (up to 9 h) broadband electromagnetic irradiation centred at 10 THz (35 fs pulses with power approx. 30 MW per pulse—average power density 1 mW cm^−2^) demonstrated altered levels of expression in 11% of screened genes which eventually led to cellular reprogramming. These two studies are another example of controversial results in the interaction of electromagnetic field with biological objects. It should be noted that even Fröhlich's theory suggests that internal long-range coherent states are important players in an object's deformation, e.g. cell division, but it does not mean it should be limited to cell division or proliferation only. Also, the theory states that effect is possible when cells are at a low level of confluency; hence, it should be expected that effects observed in the study of Williams *et al.* [53] would be rather marginal. It would be interesting to observe other parameters of cell homeostasis, like bilayer asymmetry, cytoskeleton structure and at different stages of cell division and gene expression alterations; the latter was done by Bock *et al.* [54]. Also, one of the important factors is that the quanta of energy from an exogenous source which would be capable to effectively interfere with internal long-range coherent states should be above the kilotesla level, which at 300 K corresponds to approximately 6 THz; interestingly, in a study by Bock *et al.*, this condition was met. An extended discussion on Fröhlich hypothesis and correspondent experimental evidence could be found in mini-review by Weightman [55].

### 4.3. Power versus energy

In any study, it is important to provide as much information about the source of irradiation as possible, and it should not be limited to frequency and power of the stimulus. The most common parameters which were defined in different studies as detrimental are frequency or frequency range, instant power, peak power, average power, time of exposure, repetition rate or specific absorption rate (SAR). The rationale why one or another type/mode of MMW or terahertz radiation was used in particular study varies and depends on experimental design, previous findings or theoretical conclusions. For example, Fröhlich's theory assumes the importance of the frequency of the external stimulus, due to condensation of long-range coherent states into single mode. This concept was supported by experimental observations of Grundler [56,57], in which the alterations in the growth rate of aqueous yeast culture were affected by weak 42 GHz radiation. The width of the resonance band was only 8 MHz (see also [47]). These results led to the formation of the ‘frequency window’ concept pointing to the existence of resonance effect in biological systems exposed to MMW and terahertz radiation.

A similar concept was simultaneously developed for the power applied to the sample (power window), and it was tightly connected to the observed resonance effect in *E. coli*. In particular, it was shown that the half-width of the resonance decreased from 100 to 3 MHz as the power of the stimulus was attenuated by several orders of magnitude [58]. Unfortunately, there are few if any other examples of the ‘window’ effect on different experimental models other than *E. coli*. More detailed discussion of question can be found in the review by Belyaev [58]. However, overall the power (or more precisely the power density) applied is one of the key parameters of MMW–terahertz stimulus characterization. It is because any type MMW–terahertz radiation is highly absorbed by water, and thus, the sample heating effect caused by the stimulus is an important issue in the process of MMW–terahertz effect evaluation. It was demonstrated [58] that alterations in power density applied to the sample could be compensated with reciprocal changes in the exposure duration to achieve the equivalent physiological effect. Exposure time is another important parameter and apparently not just because of the potential sample heating. It is possible that problems with reproducibility of effects from different studies could be related acute and chronic application of the exogenous electromagnetic stimulus. Williams *et al.* [53] studied the effect of MMW–terahertz on cell cultures after acute 3 h exposure, while Bock *et al.* [54] observed the effect after a much longer exposure time (9 h). Another quantitative measure commonly used for characterization of the interaction of MMW–terahertz radiation with biological samples is the SAR which represents the power absorbed by sample per unit mass which is dependent on the sample's specific conductivity. However, often, this parameter is assessed as a change in the sample's temperature per unit of exposure time, which turns SAR into heating rate characteristics of the MMW–terahertz power absorption by the tissue. Another factor which may also influence the effect of MMW–terahertz exposure is modulation of the radiation. As mentioned before, the first observations of MMW induced auditory effects revealed that only amplitude modulated signal could be perceived. Another conclusion is that the high-power pulses of MMW–terahertz radiation can have a more pronounced biological effect than continuous radiation [40,59]. Water is a strong absorber of MMW–terahertz energy due to hydrogen bond network formed between molecules; thus, the absorption spectrum of an aqueous solution containing biomolecules is dominated by water. To cause the experimentally significant level of interaction between bio-macro-molecules and MMW–terahertz radiation (which depend on the sensitivity of the instrumentation used), the power applied should be sufficiently high, while the exposure time should be kept short to prevent significant alterations in thermal equilibrium (or keep such alterations to a minimum). This way the average power applied to the sample is low and thus, undesired thermal fluctuations could be eliminated [55,60]. The inconsistency in results from different studies discussed in this review could be partly be caused by the variability in experimental designs. Kleine-Ostmann *et al.* [61] observed this and they described a methodology to identify the effects of terahertz radiation in *in vitro* experiments. For example, the experimental chamber and the sample should be electromagnetically coupled to the exposure field. The MMW and terahertz fields are often very inhomogeneous because the MMW–terahertz radiation beam and the samples' dimensions are comparable with the wavelength of the radiation. Also, the possibility of the standing wave at specific frequencies should also be considered [61]. In addition, attention should be made to the environmental factors as a source of potential errors. For example, the experimental conditions like the sample temperature, humidity, level of CO_2_ as well as the potential contaminant electromagnetic emissions from external stray sources should be monitored and controlled. To avoid unnecessary errors, the multiple repetitions of the main experiment should be performed along with the positive control experiments [61].

### 4.4. Variability of effects on different *in vitro, in situ* and *in vivo* studies

**Figure 2.**
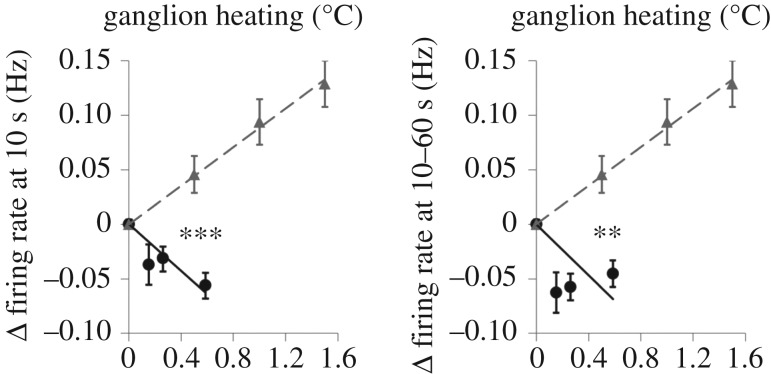
Comparison of the heating effects induced by MMW irradiation (filled circles) at three instant power density levels (represented via a sample's temperature) and by gradual bath heating (grey triangles) on changes in the subsequent AP parameters in the Retzius cells of medicinal leech: (*a*) Δfiring rate at 10 s after initiation of MMW exposure, and average Δfiring rate at 10–60 s after initiation of MMW exposure (*b*). Data are means ± standard error. **p < 0.01; ***p < 0.001 for one-tailed *t*-test comparing the MMW and bath heating effects at the heating level of 0.6°C. Linear regression lines are shown for MMW irradiation (solid) and gradual bath heating (dashed). Reproduced from [74].

Extensive studies have been done by Adey *et al.* [62], McRee & Wachtel [63], Blackman *et al.* [64] and many others [65–70] to investigate the effect of MMW on different cells and tissues as well as the whole body. One of the first studies, conducted by Wachtel on neurons of Aplysia, revealed that MMW radiation can cause suppression of neuronal activity (results showed a decrease in firing rate and in some cases full silencing of the probed neuron), and that the effect was different from the effect induced by conductive heating of the sample [71]. Changes in the firing rate of the spontaneously firing neurons, first observed by Wachtel *et al*. [71], were also seen in a variety of other studies for different frequencies in MMW range and on different experimental models. For example, the study conducted by Alekseev on BP-4 pacemaker neuron of snail Lymnaea revealed that application of 75 GHz MMW radiation of various intensities (1.8–12.6 mW at the waveguide output) caused biphasic changes in firing rate of the neuron [72]. The effect was estimated against SAR of MMW, and with highest SAR = 4200 W kg^−1^_,_ the neurons demonstrated initial 69 ± 22% decrease in spiking frequency followed by significant increase 68 ± 21% above the control value. The authors reported that the effect of MMW radiation was reproducible with equivalent heating of the neuron but also was dependent on the rate of sample heating. Interestingly, in the study, the biphasic effect of MMW was observed for exposures between 12 and 22 min, while for shorter times, only the firing rate decrease was observed. It was also noted that incubation of the neuron in Na/K-ATPase inhibitor, Ouabain, eliminated the suppressing effect of MMW. Similar results were obtained in [73] with experiments conducted on neurons in intact leech ganglia. Ganglia were subjected to 60 GHz (1–4 mW cm^−2^) MMW, and electrophysiological responses from the spontaneously spiking interneuron were compared to control ones. The study revealed a dose-dependent decrease in neurons’ spiking rate (approx. 10%) along with hyperpolarization of the membrane baseline potential and narrowing of the AP width (approx. 10%) [74,75]. Note that the duration of MMW exposure did not exceed 60 s, while the accepted US safety standard is 1 mW cm^−12^ for 6 min exposure. Hence, the effect of MMW was observed below the safety standards limits. With such a small MMW intensity used in experiments, the sample heating was small and did not exceed 1°C. Nevertheless, experiments with equivalent conductive heating demonstrated that the neurons firing rate increased (figure 2), which was opposite to what was observed under MMW [76]. The equivalent conductive heating caused narrowing of the AP, but the result was five times smaller than observed for MMW affected neurons [74]. Interestingly, in this study, the MMW caused sample heating rate was much smaller than the conductive heating while changes in the neurons electrophysiological activity more pronounced, suggesting the case was rate independent.

It is unlikely that the endogenous voltage-dependent or potential creating mechanisms would directly interact with the exogenous high-frequency stimulus. Adair's analysis of MMW interaction with voltage-dependent transmembrane channels showed that alterations in the probability of channels opening would be very low [70]. Also, the change in membrane potential is directly proportional to the intensity of the external stimulus and inversely proportional to the frequency. For power densities of approximately 10 mW m^−2^ and frequencies of approximately 10^9^–10^10^ Hz, the membrane potential shift is approximately 10^−4^ mV, which is neglectable value with respect to the natural internal fluctuations in resting membrane potential [77]. Thus, the effect of MMW on neurons is a result of coupling with another membrane-related mechanism. Studies by Ramundo-Orlando *et al.* [70,78–81] showed increased permeability in membranes of artificial liposomes upon the application MMW–terahertz radiation. Membranes loaded with carbonic anhydrase liposomes subjected to 53.37 GHz 0.1 mW cm^−2^ radiation exhibited an enhanced carbonic anhydrase reaction rate when *p*-nitrophenyl acetate was applied [79,82]. It was concluded that MMW radiation causes an increase in membrane permeability, dependent on bilayer curvature and a possible role of water molecules bound to the functional groups of lipids in the glycerol region. This conclusion is consistent with studies by Beneduci *et al.* [69,83,84], where the multilamellar vesicles (phosphocholine based) were exposed to a wide-band and various monochromatic MMW radiation (53.37, 62.10, 65 GHz) of low power density (0.0035–0.01 mW cm^−2^) for up to 4 h. It was shown that MMW induces the reduction in the water ordering in the nearest proximity to the membrane surface in time- and hydration-dependent manner. The authors also pointed on accumulation phenomena during the exposure and that absorbed electromagnetic energy stored in the form of chemical potential but not thermalized [69]. This agrees with another study conducted on mixed phospholipid monolayer exposed for up to 5 h to 60 GHz, 0.009–0.9 mW cm^−2^, showing a significant increase in lipid monolayer lateral pressure upon MMW application [85]. It was also demonstrated with fluorescence microscopy that exposure of the murine melanoma cells and Jurkat cells to 42.25 GHz with a power density up to 1.23 W cm^−2^ causes reversible externalization of phosphatidylserine [86]. Altogether these studies indicate that MMW-related effects could be mediated via interaction with cell membrane interphase structures like superficial water and/or the Guy–Chapman layers.

A decrease in neuron input resistance upon low-intensity (µW) MMW exposure was recently reported for rat cortical slices [87] and spontaneous electrical in the murine sural nerve is inhibited upon application of 45 mW cm^−2^, 42.25 GHz MMW irradiation [88]. In experiments with mice treated by anticancer chemotherapy, the utilization of MMW radiation resulted in an increased level of CD69 expression compared to the control group [89]. Sun *et al.* [90] showed that 94 GHz irradiation induced a statistically significant increase in the calcium spiking of keratinocytes, and there was facilitation of frog muscle recovery after pulse train stimulation due to the application of 42.19 GHz irradiation [91]. Changes to the compound AP were frequency independent (40–52 GHz range) and similar to the effect of a conventional temperature increase of 0.4°C [91]. Investigation of ion transport across the lipid bilayer conducted by Alekseev & Ziskin [92] revealed that 54–76 GHz MMW irradiation induced increased current flow through artificial pores in membranes by up to 5%, did not change passive membrane conductance, increased membrane capacitance by 1.2% and these results were equivalent to alterations caused by a temperature increase of 1.1°C. The diversity in results is often thought to be the result of the ‘window effect’, which assumes that the effect of MMW radiation on tissues highly depends on parameters of radiation such as MMW frequency, average and peak power/intensity of irradiation, exposure time, type and frequency of modulation, far or near-field effect and average SAR. Some studies emphasize the crucial effect of low-frequency modulation of MMW exposure: in experiments with chick brain tissue, MMW amplitude modulation with 20 Hz resulted in increased calcium release into the extracellular space [64]. Finally, variation in water content, molecular composition, interstitial liquid salinity and other parameters between experiments may also contribute to different experimental outcomes.

Other issues that need to be addressed regarding the effects of MMW radiation are blood–brain barrier (BBB) permeability changes, possible alterations in intracellular calcium homeostasis and effects on DNA–RNA-associated systems. Most studies agree that the increase in BBB permeability caused by MMW exposure in the GSM frequency range is mediated by the MMW heating effect; changes were observed at rather high SARs and albumin extravasation was reported at an SAR of more than 2.5 W kg^−1^ [93]. It is also believed that upregulation of ornithine decarboxylase by MMW radiation may cause BBB molecular damage and thus be a mediator of MMW toxicity, but again this is at exposure levels above the safety limits. Another important cell function modulator is free intracellular calcium, the smallest signalling molecule and possessing a high charge relative to its size; it involved in the regulation of a myriad of cell functions. Intracellular-free calcium concentration perturbations were observed in mouse synaptic terminals exposed non-synaptic surfaces of the plasma membrane [94]. Discovering genotoxic effects of any kind of electromagnetic radiation was always the central goal of research in the area under discussion. Massive genotoxicity studies were conducted to reveal potential risks of wireless communication technologies such as GSM, CDMA, Wi-Fi, WiGiG, etc. Nevertheless, most of the studies conclude that within the current safety limits, no detectable effects of MMW exposure are observed. In studies by Fritze *et al.* [95], a slight increase in early expression of receptor-related genes (*c-fos, c-jun*) and heat shock-related genes (*hsp72*) was observed at 900 MHz, 1.6 GHz and 2.5 GHz MMW radiation and only under conditions of high SAR (in the range of 7.5 W kg^−1^) [96,97]. For more detailed information, the reader is referred to the reviews by Hossmann & Hermann and Haarala *et al*. [98,99]. Studies considering the effect of MMW on cancerous cultures demonstrated a shift in cell metabolism rate. In particular, application of 53.57–78.33 GHz low power (approx. 1 mW cm^−2^) radiation to erythroleukaemia type K562 cells for 1 h (in four separate sessions) caused an increase in glucose metabolism via an aerobic pathway [100,101] and a significant decrease in cell proliferation. By contrast, in a subsequent study on cultured melanoma cells (RPMI 7932) exposed to 42.2 and 53.57 GHz (power densities 0.14 and 0.37 mW cm^−2^ correspondingly) for up to 4 h, no change in proliferation was observed nor was the cell cycle affected [100]. Thus, the effect of MMW strongly depends on the nature of the biological subject and the parameters of the stimulus (discussed above). More on the effect of MMW on cells in *in vitro* and *in vivo* experiments can be found in more specialized systematical reviews [91,102,103].

### 4.5. ‘High power low energy’ strategy

**Figure 3.**
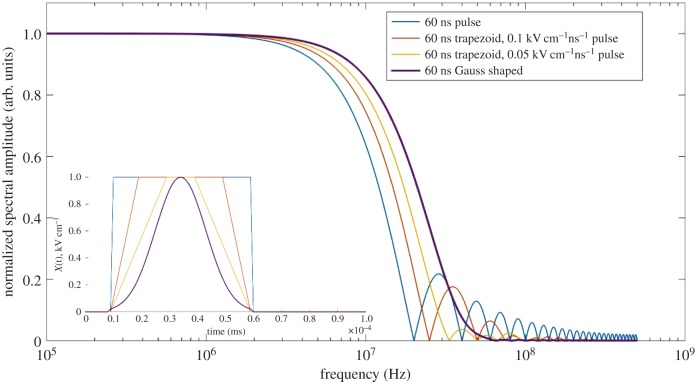
Amplitude spectra of nsPEF pulses of various shapes (speed of rising and decay front). The shapes of four different of nsPEF (60 ns, 1 kV cm^−1^) are shown in the inset. Note, the Gaussian-shaped pulse has the most monotonic spectrum.

When sources of nanosecond electrical pulses (figure 3) of extremely high intensity became available, new biological effects were revealed and this time with strong experimental and theoretical support. These sources achieve a pulse width of approximately 10–600 ns and intensity of 1–30 MV m^−1^, and are commonly referred to as nanosecond pulsed electric field (nsPEF).

One of the first studies showing the striking effect of nsPEF was done by Vernier *et al.* [104]. Application of 30 ns nsPEF with greater than 1 MV m^−1^ to Jurkat T lymphocytes caused a rapid increase in intracellular calcium by up to 25%. It is notable that application of common calcium channels blockers did not prevent alterations to calcium concentration, whereas application of thapsigargin (an agent which exhausts the caffeine-sensitive internal calcium store) reduced the effect significantly, and pretreatment with the calcium ionophore, ionomycin eliminated the effect. Schoenbach *et al*. [105–107] demonstrated that application of a 10 ns, 15 MV m^−1^ nsPEF to the same Jurkat cells affects pre-messenger RNA splicing mechanisms by increasing the level (i.e. the relative fluorescence of immunocytochemically labelled nuclear speckles) of intrachromatin granule clusters. Together, these effects indicate that nsPEF stresses internal cellular structures, including mitochondria and endoplasmic reticulum. It was also shown that the stimulus causes phosphatidylserine to exit the cell, which is a component of apoptotic signalling. Pulse stimulation also caused small but transient spikes in internal sodium concentration [104]. Later studies showed that phosphatidylserine release does not happen immediately, but that the process develops over several minutes following the stimulus, and appeared to be calcium-independent [108–114]. The initial assumption was that nsPEF causes not just intracellular organelle stress, but also cause cell membrane poration (forms ‘nanopores’); this found its support in molecular dynamics simulations conducted by Vernier. Modelling showed the formation of water bridges across the lipid membrane in the presence of a strong, transverse electric field. Experimental studies also persuasively support this idea. Studies by Ibey [115] and Pakhomov [116], carried out on cultured GH3 and CHO cells, demonstrated that the application of 600 and/or 60 ns, and 0.1–0.6 MV m^−1^ nsPEF caused a long-lasting increase in cell permeability, although the power functions for the two types of stimulus appeared to have different parameters [117].

**Figure 4.**
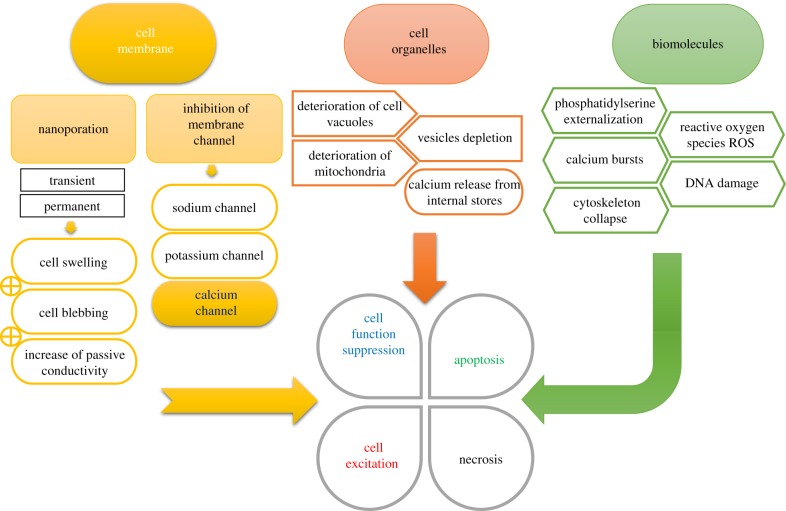
The graphic representation of diverse effects on a membrane, organelle and molecular level caused by application of nsPEF pulses to the cells. Data summarized from different studies and obtained from experiments conducted on different cell types. Thus, the involvement of a particular mechanism may vary. Also, depending on nsPEF pulse parameters, the final cell fate could be different as well, which is represented in quatrefoil. (Online version in colour.)

Formation of re-sealable nanopores in the plasma membrane could be potentially exploited for control of transmembrane transport or control of the cell excitability. This potentially could be used as a wireless tool for neurostimulation. Indeed, one study demonstrated the possibility of the neuron excitation while applying just one single nsPEF (10 ns, 27.8 kV cm^−1^) to the axon bundle. The stimulatory electrode was placed in sufficient distance from the cell bodies, where the neuronal responses were recorded with an intracellular electrode. Thus, there was no interference with between recording electrode and stimulatory nsPEF delivering electrodes [118]. It was also shown that nsPEF stress mitochondria; application of 15 ns, 8 MV m^−1^ nsPEF induced a decrease in the potential of the internal membrane of mitochondria, and the effect appeared to be calcium-dependent and developed over time after the stimulus. The mitochondria-related effect was dependent on the high-frequency component of the pulse spectrum [119]. It is particularly significant that nsPEF, demonstrated in primary cultures of rat hippocampal neurons, depolarizes the cell membrane in a dose-dependent manner and eventually evokes an AP [120]. A summary of *in vitro*, *in vivo* and *in silico* studies [105,116,120–127] is provided in figure 4.

## 5. Terahertz radiation and tissue

Owing to the high frequency of terahertz and the corresponding subpicosecond range of the transient pulse, probing of different liquids and solutions of polar and non-polar or charged solutes is possible. In the case of picosecond pulse use, its broadband spectrum allows one to investigate the sample's permittivity, conductivity, the coefficient of refraction and absorption, test multilayered materials and fit parameters of the complex Debye model for a particular sample.

The spectrum range most commonly considered to be terahertz is from 0.1 and 10 THz. However, some investigators extend this down to the lower gigahertz range and/or up to the far infrared range near 20 THz [128] or even 30 THz [54]. In more detail, terahertz radiation has a wavelength of 30 µm to 3 mm, transient times of 0.1–10 ps, wavenumber of 3.3–334 cm^−1^, single photon energy of 0.4–41 meV (or 2 × 10^−22^ to 1.3 × 10^−20^ J) and equivalent temperature range of 4.8–478 K. This energy range puts terahertz waves at the ‘bridge’ between the quantum interpretation of interactions with matter at visible light and higher energies and the continuum form of interpretation of interactions at lower energies. Transmission, reflection and absorption are important descriptive parameters as well and the most commonly used in contemporary terahertz-based analysis. Note that for biological samples, scattering of terahertz is of little significance because the wavelength is large compared to the size of biological molecules and other cell components. According to Svanberg [129], low molecular weight diatomic polar molecules have an energy gap between two rotational states of about 1 meV which corresponds to a radiation frequency of about 240–250 GHz. Similarly, the energy gap between two consecutive low-level vibrational states is about 100 meV which corresponds to a frequency of 24–25 THz of a single photon. Also, terahertz lies in the energy range of hydrogen bonds, charge transfer reactions and van der Waals interactions [54]; this means that even simple molecules absorb terahertz well. In the case of large biomolecules such as proteins, DNA, RNA, lipids and even polysaccharides, with their multiple and unique vibrational modes and complex intramolecular interactions (e.g. protein folding and DNA double-strands) and associated counter-ion aura, terahertz are expected to be a good probing tool for molecular recognition as well as a potential functional affecter. Proof of this can be found in the ever-growing body of studies showing the terahertz spectral signatures of different biomolecules such as biotin [130] DNA, albumin, collagen [131], carbamazepine, glucose, lactose anhydrate, indomethacin [132], lactose [133] and many others. Thus, terahertz could provide information on internal dynamics within large molecules in the subpicosecond range, used for characterization and potentially alteration of function. In contrast with classical techniques for the study of large biomolecules conducted with X-rays, terahertz does not have enough energy for photoionization and does not cause tissue damage. This allows the use of terahertz as a tool in ‘wet lab’ conditions and on *in vivo* samples*.*

### 5.1. Environmental and safety factor (individuals)

As has been the case for all other types of electromagnetic radiation, the question of safety and potentially harmful effects of terahertz on biological tissues was raised as soon as the sources of radiation became available. Skin is the primary recipient of terahertz applied to the individual; thus, many potential health risks are related to the skin's superficial cellular layers. Studies conducted on dermal fibroblasts exposed to 2.45 THz, 84.8 mW cm^−2^ radiation for short periods of time revealed a 3.5-fold increase in the expression of heat shock proteins, although the same results were observed in a control group treated with a heated (3°C increase) media for the same period. This showed the thermal effect of terahertz radiation. Nevertheless, the authors emphasized that despite the fact that the same rate of cell survival was observed in both experimental groups, terahertz-treated cells demonstrated increases in genotoxic controls [134]. By contrast, a study performed on different skin cell types *in vitro* with 0.380 and 2.520 THz radiation and power intensities ranging from 0.03 to 0.9 mW cm^−2^ revealed opposite effects. Cells were exposed for much longer periods of time and tests did not indicate an increase in DNA damage in the comet assay for both frequencies used [135]. Another study conducted on human lymphocytes revealed genomic instabilities caused by terahertz radiation: application of continuous-wave 0.1 THz radiation for several hours (up to 24 h) at power intensity of 0.031 mW cm^−2^ to dividing lymphocytes demonstrated the changes in chromosome number and alterations in the replication timing of their centromeres of some chromosomes; the study assumes interaction of applied stimulus with low-frequency collective vibrations of DNA molecule [136].

### 5.2. Environmental factor (plants)

Plants are also potentially subject to exposure to environmental terahertz radiation, which may, of course, have an indirect impact on humans. In experiments on rice, application of 2.5 THz and higher frequency radiation of mild power (1–10 mW cm^−2^) resulted in an increase in breeding speed, stem width, number of leaves and consequently in an increased number of grains per stem [137]. Similar studies performed with black beans [138] also revealed increased parameters of plant growth including a significant increase (25%) in the number of grains per stem for an exposure of 45 min. A recent study conducted on wheat demonstrated that exposure of the seed to 3.68 THz for 3 h resulted in a significant shortening of the seed imbibition period, an increase in the length and mass of the stem and panicle and an increase in the number of grain per stem. The effect was dependent on the quality of the seed itself and authors assumed that the effect of terahertz treatment was mediated by interaction with m-RNA and proteins of endosperm that regulate water movement into grains [139]. A study conducted on *Saccharomyces cerevisiae* yeast revealed that 150 min exposure to 0.34 THz radiation significantly enhanced the colony growth rate [140]. Overall, although the number of studies is limited, terahertz radiation appears to have a stimulatory effect on plant growth.

### 5.3. Environmental factors (animals)

Some *in vivo* studies have also been performed on animals [141]; fruit flies were exposed to 2.5 and 6.69 THz with power in the range of 1–10 mW for about 2 h; this resulted in alterations in gene expression [141,142]. Simulations predicted a total temperature increase of just 0.5°C, not a dramatic thermal shock, especially considering that during flying the active heat production of muscles may cause an increase in body temperature of 10°C or more. Terahertz radiation also affects warm-blooded animals who have body temperature control. In experiments conducted on mice exposed briefly to terahertz (3.6 THz, *λ* = 81.5 µm, 15 mW, 15 min) behavioural alterations were observed, especially in anxiety states [141,143]. In the same fashion, exposure of rats to 150.176–150.664 GHz at radiation power 4 mW and power flow 3 mW cm^−2^ for durations of up to 1 h demonstrated the development of depression in the tested animals [141,144]. The same group later designed more complex experiments and used experimental animals with induced hypokinetic stress, after conditioning animals were subjected to the same terahertz radiation protocol but of a lower intensity. Tests showed changes in blood antioxidant activity with respect to the control group, and the authors hypothesized a key role for nitric oxide (NO) molecules as an intermediator of biological effects [145]. Utilization of label-free detection of NO in injured neurons has been done by Abbas *et al.* [146]. In the study, the medicinal leech nervous cord was slightly damaged, and change in the formation of NO was monitored by observing the changes in sample's transmission in the range of 140–220 GHz. Introduction of the nerve lesion resulted into decreased in transmission coefficient 0.8 dB comparatively with the intact tissue and along all tested frequency range. Within 15 min after the injury, some slow recovery towards initial values appeared, probably due to degradation on NO in the test chamber. An additional test with the addition of NO production blocker (L-NAME) demonstrated no significant change in transmission spectra when compared with controls.

### 5.4. Global studies

The known biological effects of terahertz radiation include cell stress, mitochondrial stress, organelle function disruption, modification of membrane permeability, DNA damage, protein modification, apoptosis, tissue coagulation, stress protein expression, chromatin perturbation, etc. The ambitious goal to investigate the potential genotoxic effects of terahertz radiation in biological tissues was at the heart of THz-BRIDGE Project [147]. The effects of different modes of radiation (CW, pulsed, modulated) as well as the potential range of hazardous radiation were also under the scope of investigators. Briefly, according to the final report, terahertz radiation at any frequency does not cause harmful changes to DNA, DNA bases, membranes, cultures of epithelial cells or human keratinocytes. However, changes in liposome permeability were reported, as well as signs of genotoxicity in lymphocytes (although experiments in whole blood did not replicate this effect). Overall, the general conclusion in the report was that terahertz radiation has no harmful effect on tissue within the stipulated safety limits of exposure. Nevertheless, as has been seen from later studies discussed in this review, new data may argue that terahertz radiation can, in fact, have a biological effect.

#### 5.4.1. Potential targets: water

**Figure 5.**
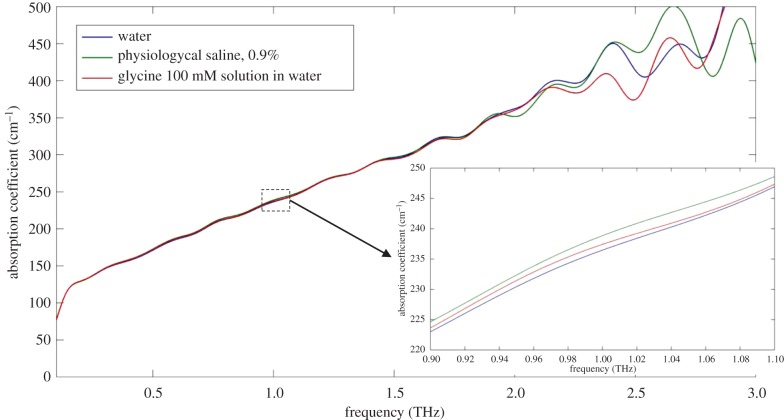
The absorption spectra in terahertz range for distilled water (blue), physiological saline —0.9% NaCl (green) and water solution of 100 mM Glycine (red). In lower terahertz range, all three spectra well overlap due to dominant absorption by water. The divergence between absorption spectra for all three samples demonstrated in the inset. Note, the presence of ‘bound’ water causes an increase in absorption.

Water content varies widely depending on tissue type and physiological state. Adipose tissue ranges from 18 to 32% water in [148], muscle is relatively stable at about 75% water [149], the average for brain tissue is about 78% [150] and for bone, it is about 10%. Water is a relatively simple polar molecule of a low molecular weight of 18 Da with a relatively large dipole moment of 1.85 D. As has been mentioned, water is capable of absorbing radiation in the terahertz range (figure 5) due to its energy states of molecular rotation and vibration [151,152]. In the liquid state, a water molecule has ‘spare’ electron pairs which could be shared with the hydrogen of another water molecule, thus forming hydrogen bond networks and allowing water to express extraordinary properties such as the coexistence of its liquid and solid phases [153].

Studies of the dielectric properties of water in the gigahertz and terahertz range by Kindt & Schmuttenmaer [154] revealed two main relaxation times of liquid water: a slow component of 8.4 ps (corresponding to a frequency of 19 GHz) and a fast component of 0.19 ps (0.84 THz) [155]. These relaxation times underlie the basis of the two-component complex Debye model of liquid water suggested by other authors [156,157]. Experimental data fitting with the use of this model showed a dramatic drop in the real part of the dielectric permittivity (from 78.4 to 4.9) along with an increase in radiation frequency. Later studies confirmed the two-component Debye model but suggested a slightly different fast relaxation time of 555.5 GHz [149,158]. Another study considering 0.15–3.72 THz absorption of saline solutions also suggested a slower second component (the ‘fast’ component) of the model of 693.1 GHz [159]. The permittivity above the resonant frequency of the water Debye model appeared to have a more consistent value among studies and was in the range of 3.3–4.1 (depending on the highest frequency reached in the study, measured in a range 100 GHz to 1 THz [149]). Theoretically, the limit permittivity is about 1.78 (the value of the refractive index of water at the frequency of the sodium D-line (589 nm) [155]), which is in agreement with studies conducted on frozen water samples which demonstrated the complex value of the refractive index to be 1.79 + 0.02*i* [160,161]. Overall, this points to the presence of further dispersion terms which are thought to be related to the additional intramolecular interactions. Indeed, the hydrogen bond dynamics of water could not be represented using permanent dipole moments because it requires induced dipole moments which reproduce inter- and intramolecular dynamics [151,162]. Further, experiments conducted by Thrane *et al.* [163] indicated that relaxation occurs without hydrogen bond breaking because this is the energetically favourable option. Comparison of the properties of ‘normal’ water and deuterium water at 1.8 THz revealed no substantial differences between the modes of two samples, suggesting a pivotal role for the oxygen atom (assigned to H-bond bend) [152]. The imaginary part of the dielectric permittivity, like its real part, decreases with increasing frequency. On the other hand, absorption by water increases with increasing frequency. The reason is that with a linear increase in frequency, the imaginary part of permittivity decreases in an exponential-like fashion, thus allowing increasing absorption with increasing frequency.

**Figure 6.**
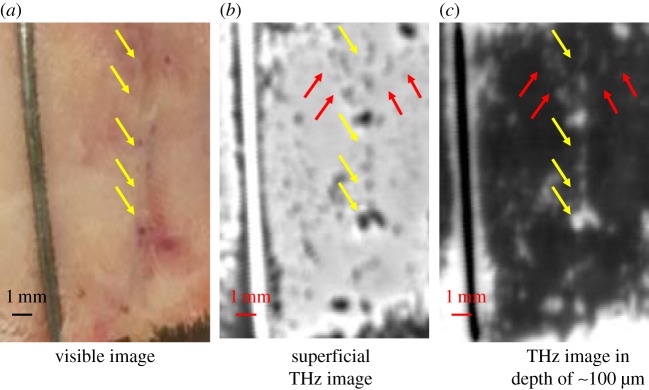
Terahertz imaging of a guinea pig skin scar (made by surgical scissors 7 days prior to imaging and sutured using surgical silk, the skin was shaved prior to imaging). (*a*) The photograph of the scar (depicted by yellow arrows), the needle was placed on the side of the photograph for orientation and terahertz contrast purposes; (*b*) terahertz imaging of scar at the superficial layer of the skin, the big dark spots along the scar are left after removal of sutures; (*c*) terahertz imaging of the scar in depth (approx. 100 µm). Red arrows indicate additional inhomogeneous formations near the scar caused by deeper tissue damage. The scan resolution is 100 µm; the images were acquired with a TeraPulse 4000 (TeraView Ltd, Cambridge, UK).

To summarize, in any kind of tissue, water is the main contrast agent for terahertz imaging [152,164,165]; this fact is the basis of terahertz radiation use for biomedical purposes. In comparison to other frequency ranges of the electromagnetic spectrum, this property offers a clear, practical advantage, like terahertz imaging of cancerous tissues, deep tissue inhomogeneity (swelling, scarring) and alteration in tissue hydration, etc. (figure 6), but at the same time, it is a disadvantage as water acts as a masking media in spectroscopy of other biomolecules of interest. On the plus side, water interactions with large biomolecules affect their sensitivity to terahertz radiation and even modify their dielectric spectrum.

#### 5.4.2. Potential targets: biomolecules

Other biomolecules like proteins, peptides, sugars, DNA and so on have a much higher molecular weight than water, although they may possess a considerably larger dipole moment. Therefore, those molecules experience the effect of the external electric field but in quite a different fashion compared to water, largely because of the great difference in the ratio of molecular mass to the total dipole moment of the individual molecule. It is obvious that dielectric properties of biomolecules vary significantly due to their complexity and variance. Thus, it makes sense to look at the basic dielectric properties of their structural blocks: amino acids and nucleonic acids.

#### 5.4.3. Potential targets: amino acids

Among the amino acids, of special interest are those having charged side chains, which is a prerequisite for interaction with electromagnetic radiation. In particular, positively charged amino acids such as arginine, histidine and lysine as well as the negatively charged glutamate acid and aspartic acid will interact significantly. Because induced dipoles in biology play an important role in a sample's dielectric properties, polar side chains are also in the group of interest as a potential contributor to the dipole moment of proteins. Serine, threonine, asparagine and glutamine amino acids belong to this group. Another group of amino acids sensitive to terahertz radiation is the aromatic amino acids, phenylalanine, tyrosine and tryptophan, due to their fluorescent properties. The dipole moments of different amino acids vary significantly; for tryptophan, it is about 3.9 D [166], for glycine 15.6 D [167] and l-arginine phosphate 32 D [168]. It appears that amino acids and their chemical variants and enantiomers are sensitive to terahertzs and show significantly varied spectrums in the terahertz range. This sensitivity is dictated by the inability of relatively large molecules to rotate at terahertz frequencies, and thus, the rotation and vibration modes of molecular components define the molecule's spectral properties [155]. In the study conducted by Matei *et al.* [169], 18 amino acids were analysed, which revealed some simple characteristics common to all tissue samples, and concluded that the absorption peaks could be grouped into five particular regions: below 6 THz, the absorption spectrum is a superposition of multiple molecular vibrations with a strong influence from hydrogen bond modes; the 6.6–8.0 THz range is dominated by COO^−^ bends; the 8.0–11.4 THz range is dominated by CCαN deformations; the 11.4–14.4 THz range is dominated by NH3^+^-group torsions; and above these, vibrations of the COO^−^-group dominate [169]. Most of the amino acids have their relaxation times in the range of 50–200 ps, although glycine, as the smallest amino acid, has a 35 ps relaxation time [170]. Specific information regarding the spectral properties of different amino acids in the terahertz range can be found in the THz-BRIDGE report [147].

An interesting example of the interaction between terahertz radiation and an amino acid is the terahertz effect on fluorescence of tryptophan (Trp) [171]. Tryptophan has a large indole side residue which consists of fused benzene and a pyrrole ring. This residue acts as a chromophore and absorbs strongly in the near-ultraviolet range. The chromophore can take one of two states with similar energies, and the fractional contribution of each state defines absorption/emission anisotropy. The important issue is that the fluorescent spectrum of Trp is highly sensitive to the surrounding environment and thus to any perturbing external electric fields. For example, irradiation of a Trp sample by 2.55 THz caused a decrease in Trp fluorescence by up to 60% [172]. The observed effect appeared to be terahertz intensity-dependent and relevant to the whole spectrum of Trp fluorescence, with an especially dramatic change in the range 320–360 nm. Terahertz radiation-induced changes in the fluorescence of Trp within a second of application reached saturation within 30 s, and cessation of irradiation lead to full fluorescence recovery within 30 s. Interestingly, the highest efficiency in Trp fluorescence quenching occurred at the same frequency as that of the highest terahertz absorption peaks for Trp. Another notable and important effect was the temperature effect on Trp fluorescence intensity: as sample temperature increase, its fluorescence decreased. Of course, the application of terahertz radiation causes sample heating, but a comparison of terahertz-induced effects (with associated heating effects) and thermally induced quenching with equivalent temperature increase alone revealed dramatic differences. For example, a 25% decrease in Trp fluorescence caused by terahertz at 25°C required an additional 65°C conductive heating-mediated temperature increase to cause the same effect. The authors hypothesize that terahertz radiation shifts the number of electrons available in the ground state for UV excitation into higher vibrational levels and, as a result, decreases the population of fluorescent molecules. Also, shifting of the UV-excited electrons into upper vibrational states may cause their non-fluorescent relaxation [172]. Further, as Trp can exist in different isomer states, it is possible that, via resonant coupling with terahertz radiation, over some transitions through a series of vibrational states, Trp can obtain a new conformation which interacts differently with UV and other environmental stimuli [30].

#### 5.4.4. Potential targets: nucleobases

Dipole moments for nucleobases, like amino acids, also deviate in their values and are significantly smaller; cytosine: 6.39 D, adenine: 2.56 D, guanine: 6.55 D, thymine: 4.31 D, uracil: 4.37 D [173]. As has been explained, the relatively low ratio of dipole moment to molecular mass determines the slow relaxation times. The rotational constants of the nucleobases along all three dimensions were all in the range of about 1–4 GHz, with the slowest time of 0.7 GHz for guanine and fastest time of 3.8 GHz for cytosine [174]. Despite the low dipole moment to molecular mass ratio, studies of the dielectric properties of nucleobases and their correspondent nucleosides in the region of 0.1–4 THz revealed their distinctive signatures in this spectral range. For example, thymine has three distinct absorption peaks at about 2.2, 2.5 and 3.3 THz; cytosine has two very wide absorption regions with approximate centres at 2.7 and 3.7 THz; whereas adenine has a more monotonous curve of spectral absorption along 2–4 THz. It was noted that absorption spectra at 300 K have rather wide resonant bands, whereas cooling the sample down to 10 K caused a split of those bands into several separated peaks with notable 10% shift in peak position towards higher frequencies, assumingly caused by the decrease in bond length at lower temperatures. Investigation of nucleosides revealed significant similarity with nucleobases, especially at low temperatures, but also revealed additional resonant bands in the region of 0.5–2 THz that could be associated with vibrational contributions from attached sugar groups [175].

#### 5.4.5. Potential targets: sugars

As terahertz radiation is sensitive to cyclic molecular structures and especially those containing benzene or pyrrole rings, monosaccharides and complex sugars, it has the potential to detect and influence them. Of particular interest among the sugars is glucose because of its obvious biological significance. With a terahertz time-domain technique, it was possible to discriminate between d-glucose, with its principal absorption peaks at 1.45 and 2.1 THz, and l-glucose, with corresponding sharp peaks at 1.45 and 2.12 THz [176]. As much as it is common for all biomolecules, hydration of the specimen leads to significant changes: the absorption curve demonstrates a monotonous increment component along the frequency range and additional resonant peaks at 1.82, 1.98 and 2.46 THz [177,178]. The authors were also able to distinguish between different anomers of the molecule. The spectral alteration that occurred in glucose monohydrate was attributed to various vibrational modes or phonon modes of the structure. It is of interest that experiments were conducted at room temperature and 13 K, but that the results at each temperature were comparable [178].

Solutions of different concentrations of glucose are easily distinguishable even at 11% concentrations (by mass) with glucose ‘fingerprints’ at 1.42 and 1.67 THz [179]. The spectra of xylose indicate low-frequency intramolecular vibrations peaks at 1.6 and 1.8 THz, the thermostable absorption peak at 2.5 THz and multiple peaks corresponding to strong intramolecular vibrations in the range 4–8 THz. Sucrose also shows two groups of absorption peaks in its spectrum: the first group has at least three well-recognizable peaks in the 1–2 THz range which soften with sample temperature increase, and the second group of multiple peaks between 2.5 and 4 THz. By contrast, d-ribose had absorption peaks in ranges around 0.5 and 1 THz but few, if any, at higher frequencies [180,181]. The two main polysaccharides groups, glycosaminoglycans and lectin binding saccharides, have very distinctive properties with respect to the terahertz absorption [59]. In particular, the mucosal heparin (as an analogue of the heparan sulfate—the anionic polysaccharide commonly present on the surface of the cell) has a typical absorption peak in the range 8.95–5.40 THz depending on what cation form of it was tested (Na, Mg, Ca, Cu, Zn). The sodium form has the highest frequency of the absorption peak, while the zinc form has the highest absorption and potassium form has the lowest. The spectrum showed the characteristic dip in the absorption, typical for all forms of the heparin, between 8 and 10 THz. By contrast, analysis of the second group of polysaccharides, in particular, dextrans of various molecular weight, revealed a much lower level of terahertz absorption compared to heparin. The heavier and more complex the poly-dextran form, the stronger the absorption. The authors concluded that high absorption properties of the heparin are a result of the incomplete occupation of the fundamental modes (conformational states) of the molecule even at room temperature. Hence, the absorbed terahertz radiation energy is not thermalized within the sample but causes the formation of new conformational states of heparin (occupation of previously vacant modes). Whereas, in the case of uncharged dextrans, all fundamental modes were already occupied and thus, there was no shift from one energy state to higher one due to the arrival of the terahertz quanta. Interestingly, the hydration properties of the dextran disaccharide and hyaluronan—two structurally similar but not identical polysaccharides—possessed remarkably similar properties in the terahertz range, based on the water dynamics in hydration layer [182].

#### 5.4.6. Potential targets: proteins

Modest combinations of two or three amino acids dramatically change the spectrum of the molecule and cannot be represented as any additive form of its components. In the case of more complex proteins, the situation evolves even further, and terahertz-mediated probing of the molecular properties is related to the secondary and tertiary structure of the protein molecule. Proteins vary considerably in size and structure, the typical size ranging from 1 to 10 nm, and dipole moments are usually determined by a few electronic charges distributed on the molecule's surface. This means that the total dipole moment of the protein molecule is small due to charge cancellation within the structure. Thus, intramolecular collective vibrations, electronic and atomic polarizations (e.g. the polar C = O bond, widely present in biology), side chain rotations on the surface of the molecule and possibly within the internal cavities, as well as long-range interactions with adjacent water molecules and counter-ions are the main contributors to the spectral signature in the terahertz range for a particular polypeptide molecule. However, as has been mentioned, for real biological solutions, water absorption dominates proteins. Thus, some methods have been developed that allow the study of proteins in solution. One of these methods is to substitute the polar water molecules with a non-aqueous solvent [183,184]. The aims and advantages of this approach are the increased solubility and access to the hydrophobic sites of the molecule, the more open structure of the protein, thermal stabilization of the enzymatic reactions and suppression of the undesired chemical activity. The main disadvantage is a dramatic loss of the natural enzymatic activity of the protein or other functions. Some tricks such as adding sugars, amino acids, crown ethers, polyelectrolyte salts and polyethylene glycols are aimed at maintaining the structure in a more natural and at, more or less, preserving the function of the protein but giving better access to the molecule itself [30]. Combined with vibrational spectroscopy, several common conformation-related features of protein structure have been studied. There are several bands in the Raman spectra: amide1 (1640–1660 cm^−1^) and amide3 (1200–1240 cm^−1^) are sensitive to the secondary structure of the molecule, while tyrosine and tryptophan markers [830 (850) cm^−1^ and 1361 cm^−1^, respectively] show sensitivity to conformational state of this molecules; conformational changes mediated by disulfide bridges are characterized by lines at 510, 525 and 540 cm^−1^ [185]. An alternative to the use of non-aqueous solvents, a low-temperature freeze technique may be used so that the response from a collective mode of many molecules can be studied. This approach is effective in studies of enzymatic activity and conformational changes. Markelz and co-workers [186] demonstrated the interaction of lysozymes with their inhibitor, *N*-acyl-glucosamine, lead to a strong decrease in the terahertz response that was caused by the loss of vibrational modes of the two-domain lysozyme structure.

#### 5.4.7. Potential targets: DNA and RNA

Similar to protein molecules, complex DNA and RNA molecules are polymers, and their dielectric properties are very different from an equal mixture of monomers. DNA molecules reach tremendous sizes, dynamically change their shape and structure and thus assumptions about DNA reactions to terahertz radiation are even less reliable. Firstly, one should keep in mind that DNA molecules can be in different condensational states. Second, because of the size, molecular motions and double-strand structure, DNA molecules do not have a permanent dipole moment. Third, the sites and mechanisms of interaction of DNA with terahertz are varied: hydrogen bonds between bases of two chains; vibrations in NH_2_ structure; hybridization complexity, affecting internal motions within the molecule; the negatively charged phosphate backbone and its associated cloud of counter-ions which cause formation of the induced dipole moment [128]; standing longitudinal acoustic modes [187]; and low-frequency vibrational modes associated with collective motions of tertiary subunits moving relative to each other [128]. Theoretical studies of the rotational and vibrational intramolecular modes of DNA predict resonant absorption lines in the terahertz range [188–190]. Studies with Li-DNA and Na-DNA in dried, well-aligned samples of molecules revealed five distinctive vibrational modes in the terahertz range, with the lowest peaks at 1.22 and 1.34 THz, respectively, which demonstrated mode softening upon sample hydration [191]. Lyophilized powder samples of DNA tested in the range of 0.06–2 THz showed that DNA absorption was not affected by the presence of polyethylene powder (used as a media in terahertz spectroscopy) and depended on the concentration of DNA in the sample. So far, the sensitivity of the DNA spectra in the terahertz range has formed a preliminary condition for developing technologies of label-free DNA analysis. In a study by Nagel *et al.* [192,193], the use of a series of terahertz wave resonators (micro-strips fabricated on a silicon substrate) with DNA deposited on it allowed discrimination of denatured DNA samples from control ones. An alternative sensor design with a terahertz resonant chamber has higher sensitivity to permittivity changes and allows differentiating between single- and double-stranded DNA. It was found to be sensitive to immobilized samples and also to the buffer of the sample. Some studies showed that application of terahertz radiation stimulated or, at least, facilitated gene expression. Alexandrov *et al.* [194] state that terahertz radiation could interact with the nonlinear, resonant breathing modes of DNA. The effect reveals itself particularly profoundly in special regions of the DNA molecule where the primary promoters of upregulated genes are located. These regions are in the ‘stand-by’ mode before the transcription process. In this mode, the separation between DNA strands forms a so-called ‘blebs’ with a length of 13 bp. Such blebs are caused by the thermally induced motions in the DNA molecule called DNA *breathing*. Molecular dynamics simulations of intrinsic double-stranded DNA breathing conducted for several genes revealed substantial variations in length, amplitude and a lifetime of the bubble [195], which suggests that terahertz have different effects on different DNA strands, regions and ultimately genes.

### 5.5. Factors of hydration

The nonlinear relationship between the protein–water solution absorption coefficient and concentration of the tested protein suggests an intense interaction of the water molecules with protein surface and enveloping layer could be as thick as tens of angstroms [196,197]. Studies with monoclonal antibodies (mAbs) conducted by Wallace *et al.* [198] demonstrated a steady increase in absorbance along terahertz range 0.25–1.5 THz. Notably, the technique was sensitive enough to detect submillimolar changes in protein concentration [198]. Also, the plot of the change in absorption coefficient versus protein concentration, measured at 1 THz, indicated the characteristic shape of gradual decrease with a subsequent plateau at some critical concentration (approx. 0.7 mM) which was explained by overlapping and sharing of individual molecules hydration layers (some 70 000 water molecules per mAbs). In some experiments, the addition of extra components into solution was done to test the extended hydration layer hypothesis, in other words, whether modification of protein hydration layer may affect its interaction with excipients. The plateau mentioned above was attenuated by the addition of 200 mM of uncharged sucrose or zwitterionic proline, whereas addition of charged arginine (same concentration) did not affect the absorption curve [198].

When studying the protein hydration shell as a crucial contributor to protein activity and structure, the protein itself has been considered to be transparent to terahertz radiation. However, in studies with a low hydration level and with frozen samples, it was demonstrated that the protein molecule contributes significantly to permittivity. In this case, the imaginary part of dielectric permittivity is a sum of products of resonant frequency with the imaginary part of permittivity for ice, bound water and the protein itself. By knowing the dielectric parameters for pure ice and the calculated contribution of the solute-bound water (only the fast component of water relaxation is important, and taking its value of 32 ps for unfrozen solute-associated water and the Arrhenius temperature dependence, it is possible to calculate the low-temperature relaxation time), one can isolate the protein component from the general terahertz response of the sample [199]. Another approach is to use spectroscopy. Sulfur atoms are heavier than carbon or oxygen and thus have their vibrational states in the terahertz range. Also, some amino acids like cysteine and methionine (homocysteine and taurine are not incorporated into proteins [200]) contain sulfur atoms and can form disulfide bridges which are critical for secondary and tertiary protein structure as well as linkage of the polypeptide chains [201]. There are three conformational states for disulfide bridges and for all three, the terahertz absorption spectra demonstrate several absorption peaks. The frequencies of the disulfide bridge stretching vibrations are very sensitive to minor variations in the dihedral angle of the bridge and thus can provide information about protein structure [202,203].

#### 5.5.1. Active interaction of terahertz radiation with biomolecules

However, the interaction of proteins with terahertz radiation is not limited to simply probing. Terahertz interactions can also modulate protein properties and states, much in the same fashion as has been described for amino acids. Studies by Cherkasova *et al.* demonstrated that the application of 3.6 THz, 10 mW radiation to lyophilized bovine serum albumin (BSA) caused a change in the intensity of the UV spectral properties and the circular dichroism spectra (CD) of protein. One hour of terahertz irradiation resulted in an augmented UV absorption in the whole UV spectrum and the intensity-dependent effect on CD. The authors also noted that the effect remained for some time after terahertz irradiation was ceased (in contrast with the effect on amino acids) and demonstrated an approximately 15% increase in the natural fluorescence of the sample. This effect was attributed to the tryptophan-134 residue, which is located on the outer surface of the molecule [204]. It was also shown that irradiation of the whey protein by 0.2 THz, 140 mW cm^−12^ radiation-induced quenching of UV fluorescence by up to 20% compared to control. The observed effect had a long stabilization period exceeding 90 s of terahertz application for both onset and recovery phases. Moreover, depending on the intensity of the probing UV pulse, the effect of the terahertz-mediated quenching could reach a state of irreversibility. The effect was irreversible if the terahertz radiation intensity was higher than 80 mW cm^−2^, but at the same time, irreversibility thresholds disappear if higher terahertz frequencies were used [30,205], at least within the experimentally available power and conditions. The quenching effect appeared at a frequency of 0.2 THz, whereas no such effect was observed in experiments with just pure Trp [172]. An interesting effect of the protein–terahertz interaction was reported in a study using one of the most famous proteins in biology, green fluorescent protein (GFP). Irradiation of a GFP sample with 0.2 THz at 120 mW cm^−2^ caused a decrease in its fluorescence of 3% with respect to the control. By contrast, 2.55 THz irradiation exhibited the opposite effect and enhanced GFP fluorescence by 5%. It is notable that terahertz radiation enhances GFP's resistance to photobleaching [30,206]. In experiments with bacteriorhodopsin [207], light-induced polarization processes in the sample revealed coherent terahertz radiation from bacteriorhodopsin with femtosecond time resolution. The conclusions based on the model simulation results showed that the effect is related to redistribution of hydrogen bonds near the retina [128]. It has also been shown that terahertz radiation could affect the enzymatic reactions and activity of proteins. For example, one study [208] showed that interactions of both alkaline phosphate with *p*-nitrophenyl-phosphate and antigen with antibody were affected (reduced enzymatic activity was observed) by 0.1 THz 80 mW m^−2^ radiation in a weak but statistically significant manner. The study also revealed that the effect was not immediate but was evident 45 min after radiation onset. Irradiation by terahertz caused alterations in enzyme velocity but no actual changes in Km, although the terahertz effect disappeared if the enzyme was immobilized prior to irradiation [208].

#### 5.5.2. Active interaction of terahertz radiation with cells

In mesenchymal stem cells (MSCs) studies, 2.52 THz radiation accelerated cell differentiation into adipocytes [194,209]. Later studies using the same experimental model showed that broadband terahertz pulses (centred at 10 THz, and at about 1 mW cm^−2^) resulted in the formation of droplet-like cytoplasmic inclusion in the exposed cells and that the effect appeared to be time-dependent [54]. By using RT–PCR, gene expression analysis indicated a more than twofold increase in the level of four of the eight selected genes, namely adiponectin, GLUT4, FABP4 and PPARg. Although the effect was detectable after 2 h of irradiation, the maximal effect was observed after 9 h of exposure. Upregulated expression of GLUT4 and adiponectin lead to differentiation into adipocytes which explain the cytoplasmic inclusions observed in experiments. Similar studies employing micro-array analysis for scanning all RNA extracted from MSC showed alterations for 20 differentially expressed genes: four genes were over-expressed and the remaining 16 were under-expressed. Moreover, the study revealed that the genes could be subdivided into three distinctive groups with respect to their sensitivity to the type of terahertz exposure (CW versus pulsed) and time of exposure and that only one gene was non-selective to the type of irradiation [195]. Note that other studies have not revealed any terahertz-mediated alterations in gene expressions. Experiments on human dermal fibroblasts conducted with low power 0.38 and 2.52 THz radiation have found no DNA strand breakage or chromosomal damage [210,211]. A recent study by Hough *et al*. [40] conducted on human skin tissue model exposed to trains (1 kHz) of intense terahertz pulses for 10 min revealed significant dysregulation of MAPK, RAS and PI3 K-AKT-related signalling pathways. Another potential effect of terahertz radiation is genome damage, possible for powerful picosecond pulses. Studies conducted on one of the histone proteins, H2A, showed increased phosphorylation of the protein in response to terahertz radiation. In addition to this, an increased level of p53 protein (a tumour suppression and cell cycle regulator) was observed [39,59]. As has been mentioned, hazardous genetic effects were part of the focus of the European THz-BRIDGE programme and had revealed chromosomal perturbations in lymphocytes in earlier studies. *In situ* hybridization techniques, studies on lymphocytes with centromere-specific probes showed that application of 0.1 THz, low power radiation (about 0.03 mW cm^−2^) had varied effects on different chromosomes, ranging from null effect to asynchronous centromere replication [136]. Later studies on human–hamster hybrid cells exposed to 0.106 THz and 0.043–4.3 mW cm^−2^ radiation also indicated that prolonged 6 h exposure caused spindle disturbances during anaphase and telophase [176].

In summary, there is now substantial evidence pointing to terahertz interactions with biological matter, ranging from simple molecules like water, ionized salts and nitric oxide, to complex biopolymers such as DNA, sugars and proteins, and to cells and whole tissues. Terahertz radiation may be used in various ways ranging from probing biological samples, defining its properties and chemical content, to medical applications for diagnostics and discriminating between healthy tissues and its pathological variants. This list will undoubtedly be expanded further to the potential use of terahertz for modification of molecules and their properties, control of enzymatic reactions and perhaps to modify transcription of proteins in biotechnology.

## Data accessibility

This article has no additional data.
